# Extent to which weight loss contributes to improving metabolic dysfunction-associated and metabolic and alcohol related/associated steatotic liver disease: a study on Japanese participants undergoing health checkups

**DOI:** 10.3389/fendo.2024.1392280

**Published:** 2024-05-08

**Authors:** Tatsuya Fukuda, Takahiro Okamoto, Takahiro Fukaishi, Akio Kawakami, Makoto Tanaka, Tetsuya Yamada, Koshiro Monzen

**Affiliations:** ^1^ Mirraza Shinjuku Tsurukame Clinic, Tokyo, Japan; ^2^ Department of Endocrinology and Metabolism, Tokyo Metropolitan Okubo Hospital, Tokyo, Japan; ^3^ Department of Molecular Endocrinology and Metabolism, Graduate School of Medical and Dental Sciences, Tokyo Medical and Dental University, Tokyo, Japan; ^4^ Koganei Tsurukame Clinic, Tokyo, Japan; ^5^ Shinjuku Tsurukame Clinic, Tokyo, Japan

**Keywords:** metabolic dysfunction-associated steatotic liver disease (MASLD), nonalcoholic fatty liver disease (NAFLD), metabolic and alcohol related/associated liver disease (MetALD), alcohol consumption, weight loss

## Abstract

**Introduction:**

The incidence of steatotic liver disease has increased in recent years. Thus, steatotic liver disease is a major public health issue in Japan. This study investigated the association between weight reduction and the remission of metabolic dysfunction-associated steatotic liver disease (MASLD)/Metabolic and alcohol related/associated liver disease (MetALD) in Japanese individuals undergoing health checkups.

**Methods:**

This retrospective observational study included 8,707 Japanese patients with MASLD/MetALD who underwent health checkups from May 2015 to March 2023. The participants were monitored for its remission at their subsequent visit. MASLD was diagnosed on abdominal ultrasonography and based on the presence of at least one of five metabolic abnormalities. The impact of body mass index (BMI) reduction on MASLD/MetALD remission was assessed via logistic regression analysis and using receiver operating characteristic curves.

**Results:**

Logistic regression analysis revealed that weight loss was significantly associated with MASLD/MetALD remission. Other factors including exercise habits and reduced alcohol consumption were significant predictors of MASLD/MetALD remission in the overall cohort and in male patients. The optimal BMI reduction cutoff values for MASLD/MetALD remission were 0.9 kg/m^2^ and 4.0% decrease in the overall cohort, 0.85 kg/m^2^ and 3.9% decrease in males, and 1.2 kg/m^2^ and 4.5% decrease in females. In participants with a BMI of 23 kg/m^2^, the cutoff values were 0.75 kg/m^2^ and 2.7% BMI reduction.

**Discussion:**

Weight reduction plays an important role in both MASLD and MetALD remission among Japanese individuals. That is, targeting specific BMI reduction is effective. This underscores the importance of targeted weight management strategies in preventing and managing MASLD/MetALD in the Japanese population.

## 1 Introduction

In recent years, there has been a significant increase in the prevalence of nonalcoholic fatty liver disease (NAFLD) worldwide, and now it is the most common chronic liver disease (1–4). This increase significantly contributes to the development of liver disease complications such as cirrhosis and liver cancer (5, 6). Furthermore, NAFLD plays an important role in not only liver health but also metabolic abnormalities, including type 2 diabetes and cardiovascular disease, which, in turn, are associated with a high mortality rate (7). Therefore, the increased incidence of NAFLD is a significant public health issue, and strategies for preventing and managing NAFLD are urgently needed.

Recently, an international panel of experts introduced a novel classification for fatty liver disease known as metabolic dysfunction-associated fatty liver disease (MAFLD) (8, 9). MAFLD is characterized by either overweight/obesity or type 2 diabetes and fatty liver. Moreover, it can be diagnosed in individuals with a normal weight who exhibit at least two metabolic abnormalities along with fatty liver. Importantly, MAFLD is a more effective identifier than NAFLD in predicting the risk of future hepatic fibrosis, atherosclerotic cardiovascular disease, chronic kidney disease, and overall mortality (9). The diagnosis of MAFLD, unlike that of NAFLD, is not influenced by alcohol consumption or viral hepatitis and is applicable across various clinical settings.

The concept of MAFLD has been accepted. However, there are reservations regarding some aspects of this condition. These concerns include the different causes of the condition, the ongoing use of the term fatty, which is viewed by many as stigmatizing, and the limitation of the population to those with at least two metabolic risk factors. Thus, these factors can potentially affect the comprehension of the natural progression of the disease (10). To address these issues, the American Association for the Study of Liver Diseases and the European Association for the Study of the Liver, in collaboration with the Asociación Latinoamericana para el Estudio del Hígado, have proposed the use of metabolic dysfunction-associated steatotic liver disease (MASLD), a new term for fatty liver disease and an alternative to NAFLD. MASLD has been redefined to include the presence of at least one of five cardiometabolic risk factors based on its diagnostic criteria, unlike MAFLD, which required the inclusion of two. The consensus decision was to change the definition of MASLD to include the presence of at least one of five cardiometabolic risk factors (10). Furthermore, among patients with steatotic liver disease meeting MASLD criteria, those with high alcohol intake (weekly intake 140-350 g female, 210-420 g male) are newly classified as metabolic and alcohol related/associated liver disease (MetALD).

Weight loss is the most effective treatment for NAFLD, and numerous studies have shown that losing weight can prevent the progression of NAFLD and, in some cases, even reverse fibrosis (11, 12). In addition, in improving NAFLD, the degree of weight loss is dose-dependent (13). Taking these findings into consideration, weight loss significantly contributes to MASLD/MetALD remission, which has some similarities with NAFLD/MAFLD. However, thus far, this aspect has not been extensively examined among Japanese individuals. Therefore, this retrospective observational study aimed to investigate the association between weight reduction and MASLD/MetALD remission using data obtained from medical checkups at three clinics.

## 2 Materials and methods

### 2.1 Study design and population

The participants who visited three clinics (Shinjuku Tsurukame Clinic, Koganei Tsurukame Clinic, and Mirraza Shinjuku Tsurukame Clinic, Tokyo, Japan) for medical checkups from May 1, 2015, to March 31, 2023, were screened for possible eligibility in this retrospective observational study. The current research included participants aged ≥ 20 years who had undergone medical checkup more than twice. In patients who had three or more medical checkups, we selected data corresponding to the period when the greatest reduction in body mass index (BMI) occurred. Each participant was monitored to determine the extent of weight change between their first and second health examinations, and improvements in MASLD status were observed. Subsequently, the participants diagnosed with MASLD during their first checkup were included in this study. None of the three aforementioned hospitals provided health guidance to patients diagnosed with MASLD.

However, individuals with a history of cancers and thyroid disorders (including Graves’ disease, Hashimoto’s disease, and chronic thyroiditis); those with an FIB-4 index of > 2.67, defined as age (years) × aspartate aminotransferase (AST; IU/L)/(platelet [10^9^/L] × (alanine aminotransferase [ALT; U/L])^1/2^ (14), which indicates suspected advanced-stage liver fibrosis; those with excessive alcohol intake (females who consume more than 350 g of alcohol per week and males who consume more than 420 g of alcohol per week); those with a history of viral hepatitis; and those with missing data were excluded.

This study was conducted in accordance with the principles of the Declaration of Helsinki. The study protocol was approved by the ethics committee of Shinjuku Tsurukame Clinic (approval no. 2,401). As the study was retrospective observational in nature, the need for a written informed consent was waived. Patients were provided with the option to opt out, with the details of the analysis made available on the hospital’s website.

### 2.2 Collection of clinical data

A standardized questionnaire was used to obtain information on medical history, current comorbidities, and lifestyle-related factors. Smoking status was classified as either current smoker or nonsmoker. Exercise habit was defined as exercise of at least 30 min per session, at least 2 days per week, for at least 1 year. Physical activity was defined as walking or performing physical activity equivalent to walking for at least 1 h per day. Fast walking was defined as walking faster than people of approximately the same age and sex. Fast eating was defined as eating at a faster rate than others. Meal just before bedtime was defined as dinner within 2 h before bedtime at least 3 times a week. Absence of breakfast was defined as missing breakfast at least 3 times a week. Good sleeping was defined as feeling well rested after sleep. Regarding alcohol consumption, the frequency of drinking was classified as daily, occasional, and rarely. The amount of alcohol consumed per drinking session, with approximately 20 g of alcohol per drink, was classified as follows: less than 1 drink, 1–2 drinks, 2–3 drinks, and ≥ 3 drinks. Based on this information, to obtain the weekly alcohol consumption, the estimated weekly alcohol consumption was calculated as follows: 1 drink defined as 20 g, 1–2 drinks as 30 g, 2–3 drinks as 50 g, and ≥ 3 units as 70 g, multiplied by 7 for daily and 2 for occasional. The alcohol consumption of patients who answered rarely was assumed to be 0 g/week. Alcohol consumption change was calculated as the amount of alcohol consumed at the second medical checkup minus the amount of alcohol consumed at the first medical checkup.

The following blood sampling data were collected after 8 h of fasting: age; sex; fasting plasma glucose (FPG), hemoglobin A1c (HbA1c), AST, ALT, gamma-glutamyl transferase (γ-GTP), triglyceride (TG), high-density lipoprotein cholesterol (HDL-C), low-density lipoprotein cholesterol (LDL-C), uric acid, serum creatinine (Cr), and hemoglobin level; estimated glomerular filtration rate (eGFR); and white blood cell and platelet count. The eGFR of Japanese patients was assessed using the following equation, as proposed by the Japanese Society of Nephrology: eGFR = 194 × serum Cr^−1.094^ × age^−0.287^[(if female) × 0.739] (15). Waist circumference (WC) was measured at the umbilical level. BMI was calculated as weight in kilogram divided by height in meters squared (kg/m^2^). In terms of comorbidities, the presence or absence of diabetes, dyslipidemia, and hypertension was determined based on self-reported data. Diabetes was defined as a diagnostic history of diabetes or use of antidiabetic agents. Dyslipidemia was defined as a diagnostic history of dyslipidemia or use of lipid-lowering agents. Hypertension was defined as a diagnostic history of hypertension or use of antihypertensive agents.

BMI reduction (kg/m^2^) was defined as the BMI at the first medical checkup minus the BMI at the second medical checkup. The reduction rate of BMI (%) was calculated as (the reduction in BMI [kg/m^2^])/BMI at the first medical checkup) × 100.

### 2.3 Ultrasound finding analysis

Abdominal ultrasonography was performed by a trained clinical laboratory technologist using the Aplio MX, Aplio 300, Aplio a450, or Aplio a550 ultrasound systems (Canon Medical Systems, Tokyo, Japan). Steatotic liver was determined, as described in a previous study (16).

### 2.4 Definition of MASLD/MetALD

MASLD was defined according to the Delphi consensus statement (10). MASLD was defined as participants with steatotic liver on abdominal ultrasonography with a weekly alcohol consumption of 70 g for females or 140 g for males or less, and at least one of the following five criteria: 1) BMI of ≥ 23 kg/m^2^ or WC of ≥ 85 cm in men or ≥ 90 cm in women (17), 2) fasting serum glucose level of ≥ 100 mg/dL or type 2 diabetes treatment, 3) blood pressure of ≥ 130/85 mmHg or specific antihypertensive drug treatment, 4) triglyceride level of ≥ 150 mg/dL or lipid-lowering treatment, and 5) HDL-C levels of ≤ 40 mg/dL in men or ≤ 50 mg/dL in women or lipid-lowering treatment. MetALD was defined as participants with steatotic liver on abdominal ultrasonography with a weekly alcohol consumption of 140-350 g for females or 210-420 g for males or less, and at least one of the aforementioned five criteria. The study endpoint was MASLD/MetALD remission at the second medical checkup. Remission of MASLD/MetALD was defined as a diagnosis of MASLD/MetALD at the first medical checkup but not at the second checkup.

### 2.5 Statistical analysis

Data were presented as mean ± standard deviation, median with interquartile range, or percentage, in accordance with the distribution characteristics of the data. To compare categorical variables, the chi-square test was used. The *t*-test was used for quantitative variables. The association between reduced BMI during follow-up and baseline covariates and MASLD remission was examined via logistic regression analysis. The odds ratio (OR) and 95% confidence interval (CI) were calculated. The selection of variables that were incorporated into the multivariate logistic regression analysis was performed with a stepwise procedure. We performed logarithmic transformations on variables including γ-GTP, TG, and alcohol consumption to normalize their distributions, applying the natural logarithm (base e). For alcohol consumption, we log-transformed the value as 1 g/week in the case of zero. For Alcohol consumption change, which could be zero or negative, we log-transformed it for everyone plus 500 g/week. This transformation was integrated into our logistic regression analysis to improve model fit and interpretability. Receiver operating characteristic (ROC) analyses were conducted to calculate the areas under the curve (AUC) of both BMI reduction (kg/m^2^) and BMI reduction rate (%) for MASLD remission. The optimal cutoff values were determined using the Youden’s index (18). P values of < 0.05 were considered statistically significant. Statistical analyses were conducted using the Statistical Package for the Social Sciences software version 21.0 (IBM Corp., Armonk, NY, the USA).

## 3 Results

This study enrolled 8,707 participants with a mean age of 49.1 ± 9.4 years, and approximately 79% of them were male. The participants underwent their second health checkups at an average of 1.3 ± 0.8 years after their first, during which the average BMI decreased by 0.7 ± 1.3 kg/m^2^. Moreover, 1,350 participants were in MASLD remission in second health checkup. **Table 1** shows the baseline clinical characteristics, comorbidities, laboratory data, and lifestyle-related parameters of all participants (n = 8,707), those without MASLD remission (stable group, n = 7,357), and those with MASLD remission (remission group, n = 1,350). The stable group was slightly older than the remission group. The stable group had a higher BMI and had a significantly less reduction in BMI than the remission group. The remission group had a significantly lower proportion of male patients and a smaller WC than the stable group. The remission group had a consistently lower prevalence of hypertension, diabetes mellitus, and dyslipidemia compared with the stable group. The FPG, TG, HDL-C, LDL-C, AST, ALT, γ-GTP, uric acid, HbA1c, and hemoglobin levels and the white blood cell count significantly differed between the stable and remission groups. In contrast, the Cr levels, eGFR, and platelet count did not differ significantly between the two groups. Regarding lifestyle-related parameters, the remission group had a significantly higher proportion of participants with exercise habits and fast walking habits than the stable group. **Supplementary Table 1** shows the baseline clinical characteristics, comorbidities, laboratory data, and lifestyle-related parameters categorized by MASLD or MetALD. Patients with MetALD patients were older and had a lower BMI than those with MASLD. Significant differences were noted in gender distribution, with a higher proportion of males in the MetALD group. Differences in hypertension, dyslipidemia, various laboratory parameters, and kidney function were also significant. Lifestyle factors differed significantly between MASLD and MetALD patients, with the latter more likely to smoke and exercise habit but less inclined to fast walking. The proportions of participants having a meal just before bedtime and reporting good sleep were different between MASLD and MetALD patients.

**Table 1:** Baseline clinical characteristics, comorbidities, laboratory data, lifestyle parameters of all subjects, those who achieved remission of metabolic dysfunction-associated steatotic liver disease (MASLD) or metabolic and alcohol related/associated liver disease (MetALD), and those who did not.

	All participants	Stable group	Remission group	
n	8707	7357	1350	
Age, y	49.1 ± 9.4	49.4 ± 9.4	48.0 ± 9.4	<0.001
Body mass index, kg/m^2^	26.2 ± 3.6	26.5 ± 3.6	24.4 ± 2.7	<0.001
BMI reduction, kg/m^2^	0.7 ± 1.3	0.5 ± 1.3	1.5 ± 1.4	<0.001
BMI reduction, %	2.7 ± 4.4	2.1 ± 4.0	6.0 ± 5.3	<0.001
Male sex	6888 (79)	5880 (80)	1008 (75)	<0.001
Waist circumference, cm	91.6 ± 8.9	92.5 ± 8.9	86.8 ± 7.3	<0.001
Waist circumference in males, cm		92.8 ± 8.7	87.4 ± 6.9	<0.001
Waist circumference in females, cm		91.2 ± 9.8	84.9 ± 8.2	<0.001
Interval between health checkups, y	1.3 ± 0.8	1.3 ± 0.8	1.3 ± 0.8	0.988
Comorbidities				
Hypertension, n (%)	1643 (19)	1471 (20)	172 (13)	<0.001
Diabetes mellitus, n (%)	514 (6)	476 (7)	38 (3)	<0.001
Dyslipidemia, n (%)	1212 (14)	1091 (15)	121 (9)	<0.001
MAFLD, n (%)	8062 (93)	6968 (95)	1094 (81)	<0.001
Laboratory data				
FPG, mg/dl	95.1 ± 21.1	96.0 ± 21.8	86.8 ± 7.3	<0.001
HbA1c, %	5.7 ± 0.8	5.7 ± 0.8	5.5 ± 0.7	<0.001
TG, mg/dl	155.8 ± 112.4	160.3 ± 112.3	131.6 ± 110.3	<0.001
HDL, mg/dl	52.8 ± 13.4	52.0 ± 13.0	56.9 ± 14.6	<0.001
HDL in males, mg/dl		50.4 ± 12.0	54.3 ± 13.3	<0.001
HDL in females, mg/dl		58.7 ± 14.8	64.4± 15.5	<0.001
LDL, mg/dl	134.3 ± 32.7	134.8 ± 32.8	131.5 ± 31.9	0.001
AST, IU/l	27.5 ± 13.9	28.1 ± 14.3	24.1 ± 11.1	<0.001
ALT, IU/l	36.3 ± 26.9	37.9 ± 27.7	27.7 ± 19.9	<0.001
γ-GTP, IU/l	57.8 ± 62.5	59.6 ± 63.5	48.0 ± 55.8	<0.001
UA, mg/dl	6.2 ± 1.4	6.3 ± 1.4	6.0 ± 1.4	<0.001
Cr, mg/dl	0.8 ± 0.2	0.8 ± 0.2	0.8 ± 0.3	0.290
eGFR, ml/min/1.73m^2^	76.5 ± 13.7	76.5 ± 13.8	76.9 ± 12.9	0.278
WBC,/µl	6188 ± 1604	6249 ± 1617	5854 ± 1487	<0.001
Hb, g/dL	15.1 ± 1.3	15.2 ± 1.3	14.8 ± 1.5	<0.001
Plt, 10^4^/µL	27.0 ± 5.9	26.9 ± 5.9	27.1 ± 5.9	0.397
SBP, mmHg	126 ± 16	126.3 ± 15.7	121.1 ± 15.1	<0.001
DBP, mmHg	80 ± 12	80.6 ± 11.7	77.1 ± 11.3	<0.001
lifestyle-related factors				
Current Smoker	2128 (24)	1826 (25)	302 (22)	0.059
Exercise habit	1803 (21)	1487 (20)	316 (23)	0.008
Physically Active	3465 (40)	2902 (39)	563 (42)	0.119
Fast walking	4526 (52)	3786 (52)	740 (55)	0.023
Fast eating	4349 (50)	3642 (50)	707 (52)	0.053
Meal just before bedtime	3738 (43)	3161 (43)	577 (43)	0.878
Absence of breakfast	2299 (26)	1970 (27)	329 (24)	0.065
Good sleeping	5555 (64)	4664 (63)	891 (66)	0.067
Daily drinking	3252 (37)	2757 (38)	495 (37)	0.573
Alcohol consumption, g/week	105.5 ± 124.5	105.5 ± 124.4	105.7 ± 124.6	0.970
Alcohol consumption change, g/week	-4.5 ± 77.9	-3.4 ± 76.1	-10.5 ± 86.7	0.002
Interval between health checkups, y	1.3 ± 0.8	1.3 ± 0.8	1.3 ± 0.8	0.988

Data are shown as mean ± SD or number (percentage). p value represents the difference between the subjects with remission and stable in means (t test), or percent (chi square test).

ALT, alanine aminotransferase; AST, aspartate aminotransferase; BMI, body mass index; Cr, creatinine; DBP, diastolic blood pressure; eGFR, estimated glomerular filtration rate; FPG, fasting plasma glucose; γ-GTP, γ-glutamyl transpeptidase; Hb, hemoglobin; HbA1c, hemoglobin A1c; HDL, high-density lipoprotein; LDL, low-density lipoprotein; MAFLD, metabolic dysfunction-associated fatty liver disease; MASLD, metabolic dysfunction-associated steatotic liver disease; PLT; platelet; SBP, systolic blood pressure; TG, triglyceride; UA, uric acid; WBC, white blood cell; WC, waist circumference.

**Table 2** shows the associations between BMI reduction during follow-up and MASLD remission based on the logistic regression models. The univariate model revealed that an increase in BMI reduction was significantly associated with MASLD remission in all participants (OR: 1.784, 95% CI: 1.665–1.835), male patients (OR: 1.834, 95% CI: 1.730–1.944), and female patients (OR: 1.542, 95% CI: 1.414–1.681), all with p-values of < 0.001. The multivariate model showed that the association between BMI reduction during follow-up and MASLD remission remained significant across all groups (OR: 2.792, 95% CI: 2.610–2.992 in the overall cohort; OR: 3.131, 95% CI: 2.879–3.406 in male patients; and OR: 2.254, 95% CI: 2.001–2.539 in female patients). Lower baseline BMI and younger age were consistently associated with MASLD remission across all groups. Dyslipidemia was significantly and negatively associated with MASLD remission in all participants (OR: 0.744, p = 0.014) and in males (OR: 0.744, p = 0.033), however, no sufficiently significant associations of dyslipidemia with endpoints were detected in females. Lower FPG, Log-TG, and ALT levels were associated with MASLD remission across all groups. Higher HDL-C levels were significantly associated with MASLD remission in all participants and male patients, but not in female patients. With regard to lifestyle-related parameters, exercise habit was significantly associated with MASLD remission in all participants and in male patients. The absence of breakfast was associated with a decreased risk of MASLD remission only in male patients.

**Table 2:** Logistic regression models examining the associations of body mass index reduction in follow-up with the remission of metabolic dysfunction-associated steatotic liver disease (MASLD).

	All participants			Male (n=6888)			Female (n=1819)		
	OR	95%CI	*p*	OR	95%CI	*p*	OR	95%CI	*p*
Univariate model									
BMI reduction, kg/m^2^	1.784	1.665-1.835	<0.001	1.834	1.730-1.944	<0.001	1.542	1.414-1.681	<0.001
Multivariate model									
BMI reduction, kg/m^2^	2.792	2.610-2.992	<0.001	3.131	2.879-3.406	<0.001	2.254	2.001-2.539	<0.001
Baseline BMI, kg/m^2^	0.700	0.678-0.722	<0.001	0.753	0.713-0.796	<0.001	0.724	0.686-0.763	<0.001
Age, y	0.969	0.961-0.978	<0.001	0.978	0.968-0.987	<0.001	0.966	0.948-0.984	<0.001
Dyslipidemia	0.744	0.587-0.942	0.014	0.744	0.566-0.977	0.033	NS		
FPG, mg/dl	0.986	0.981-0.991	<0.001	0.987	0.981-0.992	<0.001	0.983	0.972-0.996	0.008
Log-TG	0.361	0.255-0.513	<0.001	0.457	0.307-0.681	<0.001	0.268	0.128-0.563	0.001
HDL, mg/dl	1.007	1.001-1.012	0.018	1.010	1.003-1.017	0.007	NS		
ALT, IU/l	0.981	0.977-0.985	<0.001	0.981	0.977-0.986	<0.001	0.969	0.949-0.989	0.003
Log-γ-GTP	NS	NS	NS	NS	NS	NS	0.369	0.187-0.730	0.004
eGFR, ml/min/1.73m^2^	0.992	0.986-0.997	0.004	NS			0.986	0.975-0.996	0.012
SBP, mmHg	0.992	0.987-0.996	<0.001	0.983	0.976-0.991	<0.001	NS		
Exercise habit	1.264	1.074-1.487	0.005	1.239	1.029-1.492	0.024	NS		
Absence of breakfast	NS			0.798	0.662-0.962	0.018	NS		
Log-Alcohol consumption change	0.374	0.208-0.675	0.001	0.356	0.192-0.663	0.001	NS		
Hb, g/dl	NS			NS			0.892	0.799-0.996	0.011

ALT, alanine aminotransferase; BMI, body mass index; eGFR, estimated glomerular filtration rate; FPG, fasting plasma glucose; Hb, hemoglobin; HDL, high-density lipoprotein; LDL, low-density lipoprotein; MASLD, metabolic dysfunction-associated steatotic liver disease; SBP, systolic blood pressure; TG, triglyceride; γ-GTP, gamma-glutamyl transferase.

**Figure 1:**
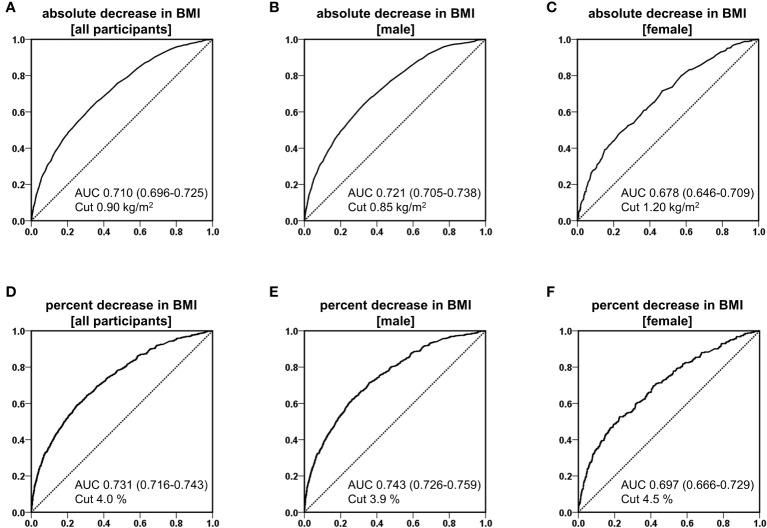
Receiver operating characteristic (ROC) curves of reduction of body mass index (BMI) for predicting the remission of metabolic dysfunction-associated steatotic liver disease in analysis of absolute decrease in BMI of all participants **(A)**, male **(B)**, and female **(C)**, and analysis of percent decrease in BMI of all participants **(D)**, male **(E)**, and female **(F)**.

**Figure 1** shows the ROC curves examining the association between BMI reduction (kg/m^2^) and BMI reduction rate (%) and MASLD remission in all participants, male patients, and female patients, with AUC and optimal cutoff values. The AUC for BMI reduction (kg/m^2^) was 0.710 in all participants, 0.721 in male patients, and 0.678 in female patients, with the optimal cutoff values of 0.90, 0.85, and 1.20 kg/m^2^, respectively (**Figure 1A–C**). The AUCs of BMI reduction rate (%) were 0.731 in all participants, 0.743 in male patients, and 0.697 in female patients, with the appropriate cutoff values of 4.0%, 3.9%, and 4.5%, respectively (**Figure 1D–F**).

**Supplementary Figure 1** depicts the ROC curves examining the association between BMI reduction (kg/m^2^) and BMI reduction rate (%) and MASLD/MetALD remission in patients with a BMI of < 23 kg/m^2^ (n = 1,214) and those with a BMI of ≥ 23 kg/m^2^ (n = 7,495), with AUC and optimal cutoff values. The AUCs for BMI reduction (kg/m^2^) were 0.669 in participants with a BMI of < 23 kg/m^2^ and 0.751 in those with a BMI of ≥ 23 kg/m^2^, with optimal cutoff values of 0.75 and 0.90 kg/m^2^, respectively (**Supplementary Figure 1A, B**). The AUCs for BMI reduction rate (%) were 0.671 for participants with a BMI of < 23 kg/m^2^ and 0.767 for those with a BMI of ≥ 23 kg/m^2^, with optimal cutoff values of 2.7% and 4.0%, respectively (**Supplementary Figure 1C, D**).

**Table 3** shows the logistic regression models of the association between BMI reduction (kg/m^2^) and improvement in steatotic liver disease in MASLD and MetALD patients separately. In individuals with MASLD, the univariate model revealed that an increase in BMI reduction was significantly associated with MASLD (OR: 1.736, 95% CI: 1.643–1.834, p < 0.001), and the association was consistent after adjustment for other covariates in multivariate model (OR: 2.822, 95% CI: 2.608–3.054, p < 0.001). Similarly in individuals with MetALD, the univariate model revealed that an increase in BMI reduction was significantly associated with MASLD (OR: 1.813, 95% CI: 1.630–2.017, p < 0.001), and the association was consistent after adjustment for other covariates in multivariate model (OR: 2.793, 95% CI: 2.426–3.217, p < 0.001).

**Table 3:** Logistic regression models examining the associations of body mass index reduction in follow-up with the remission of metabolic dysfunction-associated steatotic liver disease (MASLD) or metabolic and alcohol related/associated liver disease (MetALD).

	MASLD (n=6418)			MetALD (n=2289)		
	OR	95%CI	*p*	OR	95%CI	*p*
Univariate model						
BMI reduction, kg/m^2^	1.736	1.643-1.834	<0.001	1.813	1.630-2.017	<0.001
Multivariate model						
BMI reduction, kg/m^2^	2.822	2.608-3.054	<0.001	2.793	2.426-3.217	<0.001
Baseline BMI, kg/m^2^	0.683	0.659-0.708	<0.001	0.717	0.675-0.761	<0.001
Age, y	0.968	0.959-0.977	<0.001	0.980	0.964-0.996	0.014
Dyslipidemia	0.744	0.565-0.979	0.034	NS		
HbA1c, %	NS			0.470	0.347-0.636	<0.001
Log-TG, mg/dl	0.273	0.182-0.410	<0.001	0.392	0.225-0.683	0.001
Log-γ-GTP	0.665	0.461-0.958	0.028	NS		
HDL, mg/dl	1.007	1.000-1.014	0.049	NS		
ALT, IU/l	0.983	0.978-0.988	<0.001	0.982	0.974-0.991	<0.001
eGFR, ml/min/1.73m^2^	0.990	0.984-0.997	0.004	NS		
SBP, mmHg	0.994	0.988-0.999	0.029	0.975	0.964-0.987	<0.001
Exercise habit	1.289	1.058-1.569	0.012	NS		
Absence of breakfast	NS			0.689	0.510-0.930	0.015
Log-Alcohol consumption	1.115	1.012-1.228	0.028	NS		
Log-Alcohol consumption change	NS			0.317	0.168-0.600	<0.001

ALT, alanine aminotransferase; BMI, body mass index; eGFR, estimated glomerular filtration rate; FPG, fasting plasma glucose; HbA1c, hemoglobin A1c; HDL, high-density lipoprotein; LDL, low-density lipoprotein; MASLD, metabolic dysfunction-associated steatotic liver disease; SBP, systolic blood pressure; TG, triglyceride.

**Supplementary Table 2** shows AUC in ROC curves of reduction of BMI for predicting the remission of MASLD, and MetALD. In participants with MASLD, the AUC for BMI reduction (kg/m^2^) was 0.716 with the optimal cutoff values of 0.85 kg/m^2^, and the AUC for BMI reduction rate (%) was 0.737 with the optimal cutoff values of 3.5%. In participants with MetALD, the AUC for BMI reduction (kg/m^2^) was 0.695 with the optimal cutoff values of 0.75 kg/m^2^, and the AUC for BMI reduction rate (%) was 0.713 with the optimal cutoff values of 3.0%.

## 4 Discussion

This study aimed to elucidate the extent to which weight loss contributes to MASLD/MetALD remission among Japanese participants undergoing health checkups. Results showed that a decrease in BMI was significantly associated with the remission of MASLD/MetALD regardless of sex. Therefore, weight loss can be an important factor in managing MASLD/MetALD. Further, it was significantly associated with MASLD/MetALD improvement in patients with a BMI of < 23 kg/m^2^. Hence, weight loss can be useful in improving MASLD/MetALD even in lean individuals. Weight loss also contributed independently to their improvement in each of the MASLD and MetALD patients, respectively.

Weight loss plays a significant role in improving steatotic liver disease. In studies on patients with NAFLD, a 5% reduction in BMI was associated with a 25% decrease in liver fat content (19). Similarly, patients with NASH who had a weight loss of > 5% had a higher rate of NASH resolution and a significant improvement in the nonalcoholic steatotic liver disease activity score compared with those with a weight loss of < 5% (20). Considering these findings, a weight loss of approximately 5% can be a strong contributing factor for MASLD/MetALD remission. Several patients with MAFLD and NAFLD overlap, and weight loss may similarly contribute to remission. Indeed, Hasegawa Y et al. reported that weight loss led to MAFLD remission after 5 years in 3,309 Japanese individuals who underwent health checkups, with a cutoff value of 1.9 kg or 3.1% reduction (21). Most patients with MASLD/MetALD and MAFLD overlap. In our study, 93% of patients diagnosed with MASLD/MetALD met the diagnostic criteria for MAFLD. Similar to the results of Hasegawa Y et al., weight loss was found to independently contribute to MASLD/MetALD remission. The cutoff value for weight loss is a reduction of 0.9 kg/m^2^ in BMI or a 4.0% decrease in BMI. Our results can be useful in setting target values for weight loss in Japanese patients diagnosed with MASLD. MetALD is a condition where the effects of a certain amount of alcohol overlap with those observed in MASLD patients (10), therefore, it was hypothesized that weight loss would also be an effective strategy for remission in MetALD patients, similar to those with MASLD. As expected, our findings indeed indicate that weight loss leads to remission in MetALD patients, just as it does in MASLD patients. Furthermore, we demonstrated that the appropriate cutoff value for BMI reduction in MetALD patients is roughly equivalent to that in MASLD patients (0.75 vs 0.85 kg/m^2^). This suggests that weight management strategies could be universally beneficial across both conditions, underscoring the importance of weight loss in the therapeutic approach for steatotic liver diseases associated with metabolic dysfunctions and alcohol consumption.

Exercise therapy improves NAFLD independent of weight loss based on meta-analyses (22), therefore, it might contribute to MAFLD improvement. Consistent with these results, our study showed that exercise habit, defined as exercise for at least 30 min per session, at least twice a week, was significantly and independently associated with MASLD/MetALD improvement during the next health checkup in the overall cohort and male participants. Therefore, exercise habit can be beneficial for improving MASLD in Japanese patients similar to those with steatotic liver diseases, including NAFLD and MAFLD. However, further research should be performed to determine the usefulness of exercise for MASLD remission in female Japanese patients.

Numerous studies have reported that alcohol consumption, even in small quantities, contributes to the progression of liver diseases, including NAFLD, in a dose-dependent manner. Moreover, there is a potential for a synergistic adverse effect if there is a co-existence of metabolic syndrome and excessive alcohol consumption (23–26). Consequently, it was hypothesized that alcohol use can be an inhibitory factor in MASLD/MetALD improvement. In the overall cohort and male patients, a decrease in alcohol consumption was significantly associated with MASLD/MetALD improvement. However, this association was not observed in female participants, and baseline alcohol consumption was not associated with MASLD improvement, regardless of sex. Based on these findings, in addition to weight reduction, alcohol reduction may contribute to MASLD/MetALD improvement in men with MASLD/MetALD who consume alcohol to a certain extent. Nevertheless, both baseline alcohol consumption and reduction in alcohol intake during the follow-up period were not associated with their remission in the sensitivity analysis of MASLD and MetALD patients. In the current study, we prioritized registering cases in which weight loss was achieved and examined the association between weight loss and MASLD reduction, therefore, it is possible that the association between alcohol intake and changes in alcohol intake and MASLD/MetALD has not been accurately investigated. Further research should be performed to determine the extent to which alcohol reduction contributes to MASLD/MetALD improvement.

In the new consensus statement, patients with a certain amount weekly alcohol consumption (up to 140 g/week for females and 210 g/week for males) who fulfill the criteria for both MASLD and alcohol-related fatty liver disease (ALD) are classified as having MetALD (10). MASLD and ALD share similar histological features, including accumulation of triacylglycerol-rich lipid droplets within hepatocytes, and share common molecular alterations. Notably, an increase in the expression of cytokine IL-6, which is involved in inflammation, has been implicated in promoting the progression of both MASLD and ALD, primarily via mitochondrial dysfunction (27, 28). Given these findings, weight loss may aid in improving conditions, even in patients predominantly affected by ALD, such as those diagnosed with MetALD. Our data demonstrates that weight loss independently ameliorates both MASLD and MetALD conditions, suggesting that weight loss counseling, in conjunction with alcohol reduction advice, may be beneficial for patients with MetALD.

Patients with NAFLD who have a normal weight can exhibit the same level of fibrosis as patients with NAFLD who have obesity (29). Some reports have recommended that patients with NAFLD who are nonobese may have a higher incidence of type 2 diabetes and a higher fat percentage than patients with NAFLD who are obese (30, 31). Moreover, numerous studies have revealed that weight loss contributes to NAFLD remission in patients who are nonobese. Therefore, weight reduction might also contribute to MASLD/MetALD remission in patients who are nonobese. Our study reported that weight loss could possibly be a factor for MASLD/MetALD remission in patients who are nonobese. Further, the optimal cutoff value for weight reduction in patients with MASLD/MetALD who are nonobese is a decrease of 0.75 kg/m^2^ or a 2.7% decrease in BMI.

The current study had several limitations that must be considered. First, it included data collected from health checkups at three clinics, thereby limiting the generalizability of our findings. In addition, the participants were Japanese; thus, the applicability of our results to other races has not been confirmed. Second, MASLD/MetALD was diagnosed on ultrasonography. However, this procedure underestimates the prevalence of hepatic steatosis if there is < 20% steatosis (32). Moreover, it is completely impractical to perform a biopsy, which is the gold standard for diagnosis, in all patients suspected of steatotic liver disease who are undergoing health checkups. Third, we were unable to use factors that could potentially influence the progression of MASLD/MetALD, such as insulin resistance and inflammation, as explanatory variables. Fourth, the present study had a retrospective design, and there may be some selection bias. Further large prospective studies are needed to confirm the effect of weight loss on remission of MASLD/MetALD.

## 5 Conclusion

This retrospective observational study showed that weight reduction significantly contributed to MASLD/MetALD remission in participants who underwent health checkups. The optimal cutoff values for weight loss were a decrease of 0.9 kg/m^2^ in BMI or a 4% decrease in BMI in the overall cohort, a decrease of 0.85 kg/m^2^ in BMI or a 3.9% decrease in BMI in male patients, and a decrease of 1.2 kg/m^2^ in BMI or a 4.5% decrease in BMI for female patients. In patients with a BMI of < 23 kg/m^2^, the optimal cutoff value for weight loss was a decrease of 0.75 kg/m^2^ in BMI or a 2.7% decrease in BMI. In patients with MASLD, the optimal cutoff value for weight loss was a decrease of 0.85 kg/m^2^ in BMI or a 3.5% decrease in BMI. In patients with MetALD, it was 0.75 kg/m^2^ in BMI or a 3.0%, respectively.

## Data availability statement

The raw data supporting the conclusions of this article will be made available by the authors, without undue reservation.

## Ethics statement

The studies involving human participants were reviewed and approved by the ethics committee of Shinjuku Tsurukame Clinic (approval no. 2,401). The studies were conducted in accordance with the local legislation and institutional requirements. Written informed consent for participation was not required from the participants or the participants’ legal guardians/next of kin in accordance with the national legislation and institutional requirements.

## Author contributions

TFuku: Writing – review & editing, Writing – original draft, Visualization, Methodology, Investigation, Formal analysis, Data curation, Conceptualization. TO: Writing – review & editing, Validation, Supervision. TFuka: Writing – review & editing, Validation, Supervision. AK: Writing – review & editing, Validation, Supervision. MT: Writing – review & editing, Validation, Supervision. TY: Writing – review & editing, Software, Project administration, Funding acquisition. KM: Writing – review & editing, Visualization, Supervision, Project administration.
