# Supplementary material for: Extent to which weight loss contributes to improving metabolic dysfunction-associated and metabolic and alcohol related/associated steatotic liver disease: a study on Japanese participants undergoing health checkups
Source: Front Endocrinol (Lausanne). 2024 May 8;15:1392280. doi: 10.3389/fendo.2024.1392280 (PMC11109399; doi:10.3389/fendo.2024.1392280)
Supplement: Supplementary file 1 [file DataSheet_1.docx]

**Supplementary Figure 1. Receiver operating characteristic (ROC) curves of reduction of body mass index (BMI) for predicting the remission of metabolic dysfunction-associated steatotic liver disease (MASLD) or metabolic and alcohol related/associated liver disease (MetALD), in analysis of absolute decrease in BMI of participants with BMI < 23kg/m^2^ (A), and those with BMI ≥ 23kg/m^2^ (B), and analysis of percent decrease in BMI of participants with BMI < 23kg/m^2^ (C), and those with BMI ≥ 23kg/m^2^ (D).**

**
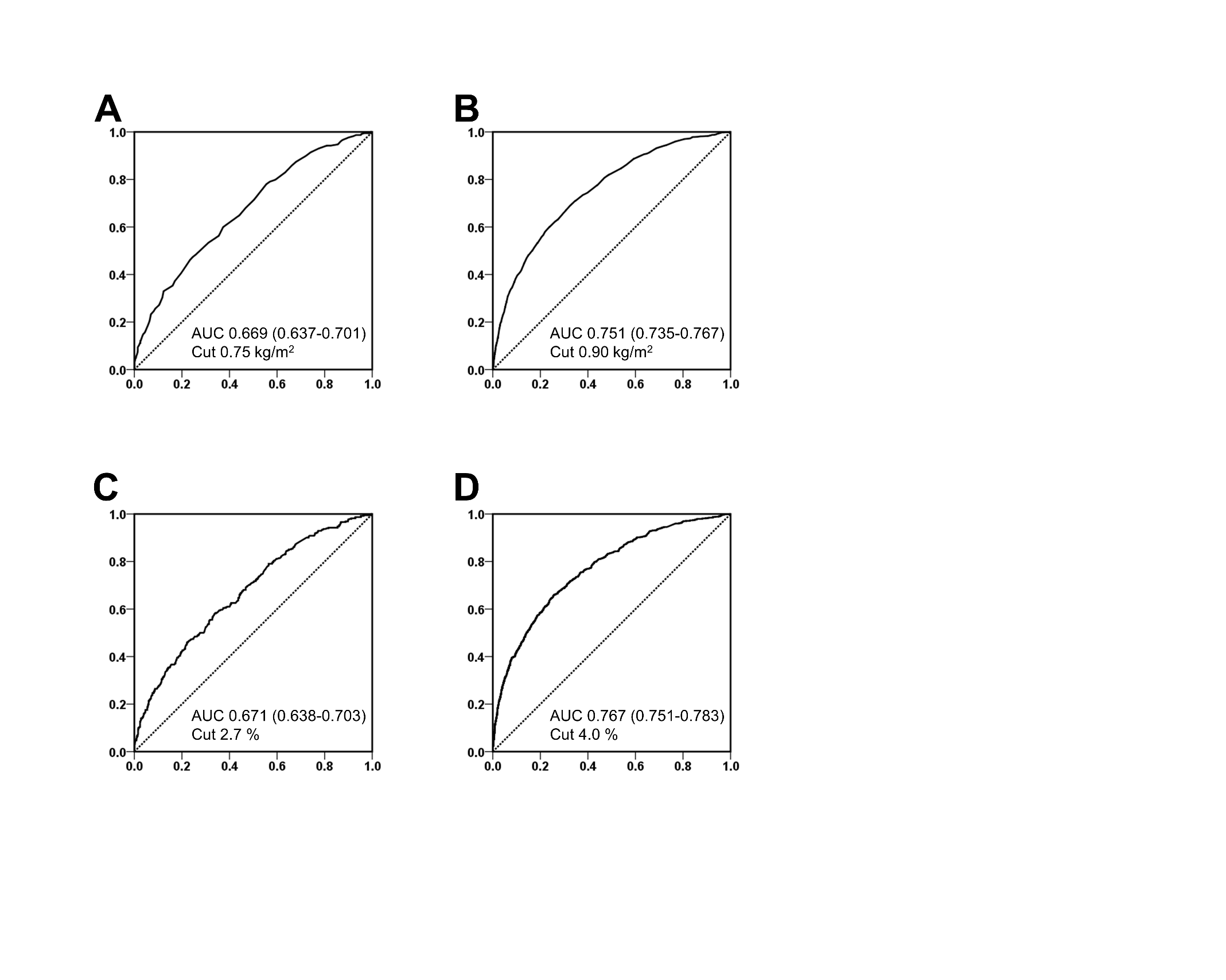
**

**Supplementary Table 1. Baseline clinical characteristics, comorbidities, laboratory data, lifestyle parameters** **of all subjects, those who achieved remission of metabolic dysfunction-associated steatotic liver disease (MASLD) or metabolic and alcohol related/associated liver disease (MetALD), and those who did not.**

|  | **MASLD** | **MetALD** |  |
| --- | --- | --- | --- |
| n | 6418 | 2289 |  |
| Age, y | 48.6 ± 9.5 | 50.7 ± 9.0 | <0.001 |
| Body mass index, kg/m^2^ | 26.4 ± 3.7 | 25.6 ± 3.1 | <0.001 |
| BMI reduction, kg/m^2^ | 0.8 ± 1.4 | 0.7 ± 1.1 | 0.020 |
| BMI reduction, % | 2.8 ± 4.5 | 2.6 ± 4.1 | 0.066 |
| Male sex | 4857 (76) | 2031 (89) | <0.001 |
| Waist circumference, cm | 91.8 ± 9.2 | 90.9 ± 8.2 | <0.001 |
| Hypertension | 1062 (17) | 581 (25) | <0.001 |
| Diabetes mellitus | 385 (6) | 129 (6) | 0.527 |
| Dyslipidemia | 922 (14) | 290 (13) | 0.044 |
| FPG, mg/dl | 94.5 ± 21.5 | 96.7 ± 19.9 | <0.001 |
| HbA1c, % | 5.7 ± 0.8 | 5.6 ± 0.7 | <0.001 |
| TG, mg/dl | 150.6 ± 102.3 | 170.5 ± 135.7 | <0.001 |
| HDL, mg/dl | 51.0 ± 12.4 | 57.6 ± 14.8 | <0.001 |
| LDL, mg/dl | 135.2 ± 35.3 | 131.7 ± 33.5 | 0.001 |
| AST, IU/l | 26.9 ± 13.6 | 29.1 ± 14.6 | <0.001 |
| ALT, IU/l | 37.2 ± 27.8 | 33.9 ± 23.9 | <0.001 |
| γ-GTP, IU/l | 48.7 ± 49.3 | 82.7 ± 84.7 | <0.001 |
| UA, mg/dl | 6.2 ± 1.4 | 6.5 ± 1.4 | <0.001 |
| Cr, mg/dl | 0.8 ± 0.2 | 0.8 ± 0.1 | 0.679 |
| eGFR, ml/min/1.73m^2^ | 76.3 ± 13.8 | 77.2 ± 13.2 | 0.006 |
| WBC, /µl | 6280 ± 1619 | 5929 ± 1532 | <0.001 |
| Hb, g/dL | 15.1 ± 1.4 | 15.2 ± 1.2 | <0.001 |
| Plt, 10^4^/µL | 27.2 ± 6.0 | 26.4 ± 5.8 | <0.001 |
| SBP, mmHg | 124.7 ± 15.9 | 127.8 ± 15.2 | <0.001 |
| DBP, mmHg | 79.1 ± 11.8 | 82.9 ± 11.1 | <0.001 |
| Current Smoker | 1442 (23) | 686 (30) | <0.001 |
| Exercise habit | 1237 (19) | 566 (25) | <0.001 |
| Physically Active | 2521 (39) | 944 (41) | 0.100 |
| Fast walking | 3198 (50) | 1328 (58) | <0.001 |
| Fast eating | 3229 (50) | 1120 (49) | 0.256 |
| Meal just before bedtime | 2488 (39) | 1250 (55) | <0.001 |
| Absence of breakfast | 1595 (25) | 704 (31) | <0.001 |
| Good sleeping | 4024 (63) | 1531 (67) | <0.001 |
| Daily drinking | 963 (15) | 2289 (100) | <0.001 |
| Alcohol consumption, g/week | 40.8 ± 40.6 | 286.9 ± 98.7 | <0.001 |
| Alcohol consumption change, g/week | 7.8 ± 51.6 | -39.0 ± 118.3 | <0.001 |
| Interval between health checkups, y | 1.3 ± 0.8 | 1.3 ± 0.8 | 0.413 |
| MAFLD, n (%) | 5987 (93) | 2075 (91) | <0.001 |

**Supplementary Table 2. Area under the curves (AUC) in receiver operating characteristic (ROC) curves of reduction of body mass index (BMI) for predicting the remission of metabolic dysfunction-associated steatotic liver disease (MASLD) or metabolic and alcohol related/associated liver disease (MetALD).**

|  | **AUC** | **95%CI** | **Cut off value** |
| --- | --- | --- | --- |
| **MASLD** |  |  |  |
| absolute decrease in BMI | 0.716 | 0.698-0.733 | 0.85 kg/m^2^ |
| percent decrease in BMI | 0.737 | 0.720-0.754 | 3.5 % |
| **MetALD** |  |  |  |
| absolute decrease in BMI | 0.695 | 0.666-0.724 | 0.75 kg/m^2^ |
| percent decrease in BMI | 0.713 | 0.685-0.742 | 3.0 % |
